# Case Report: Durable partial response to icotinib plus crizotinib in a lung adenocarcinoma patient with double uncommon *EGFR* G719D/L861Q mutations and an acquired novel *CUX1*-*MET* fusion

**DOI:** 10.3389/fonc.2022.911362

**Published:** 2022-07-26

**Authors:** Lanzi Ou, Yicong Tang, Yanming Deng, Lijie Guo, Qingqing He, Tingting He, Weineng Feng

**Affiliations:** ^1^ Oncology, Guangdong Medical University, Zhanjiang, China; ^2^ Department of Head and Neck/Thoracic Medical Oncology, The First People’s Hospital of Foshan, Foshan, China; ^3^ Medical Department Shanghai OrigiMed Co., Ltd, Shanghai, China

**Keywords:** lung adenocarcinoma, *EGFR* mutations, *CUX1*-*MET* fusion, icotinib plus crizotinib, partial response

## Abstract

Non-small cell lung cancer (NSCLC) patients harboring *MET* exon 14 skipping or high *MET* amplification display a high rate of response to MET inhibitors. However, *MET* fusions in NSCLC have rarely been revealed. In this report, a 63-year-old woman with lung adenocarcinoma (LADC), harboring *EGFR* exon 18 G719D and exon 21 L861Q mutations, received first-generation, *EGFR*-tyrosine kinase inhibitor (TKI) icotinib therapy. Next generation sequencing (NGS) results only displayed an *EGFR* T790M point mutation following icotinib resistance. Thus, the patient was treated with osimertinib and achieved a stable disease (SD). However, disease progressed after 15 months and a novel *MET* fusion (*CUX1* exon14-*MET* exon15) in addition to *EGFR* G719D/L861Q mutations were simultaneously detected in a tissue biopsy sample. After more than nine months, the patient subsequently achieved a PR with the combination of icotinib and crizotinib. To our knowledge, this is the first case of LADC patient displaying the presence of *EGFR* double uncommon mutations and an acquired novel *CUX1*-*MET* fusion that has benefited from icotinib plus crizotinib treatment. Following nine months of PR with icotinib plus crizotinib, the patient, until the time of publication, is exhibiting stable disease. The results suggest that the *CUX1*-*MET* fusion may be sensitive to crizotinib, although previous reports indicated that some *MET* fusion cases did not respond to crizotinib. Given this disparity, distinguishing *MET* fusion partners when crizotinib is used in LADC treatment is also very important.

## Introduction

Mutations within the epidermal growth factor receptor (*EGFR*) are an important driver of lung cancers. Targeting *EGFR* with tyrosine kinase inhibitors (TKIs) is the main precision medicine treatment option and the standard treatment for lung adenocarcinoma (LADC) patients harboring *EGFR* activating mutations (1, 2). *EGFR*-TKIs have markedly improved the prognoses of patients with non-small cell lung cancer (NSCLC) harboring *EGFR*-activating mutations. Mutations such as *EGFR* 19del and *EGFR* L858R are known as “classical mutations” that often confer a better response to *EGFR*-TKIs (3, 4). Other mutations within *EGFR* exons 18-21 such as *EGFR* L861Q and G719D mutations, which are non-classical mutations with a low incidence, a poor response, and an uncertain resistance when treated with an *EGFR*-TKI, have also been reported (5). In recent years, several *EGFR*-TKIs that are used as a first-line treatment for NSCLC with *EGFR* mutations, such as the first-generation TKIs erlotinib, gefitinib and icotinib, second-generation TKIs afatinib and dacotinib, and third-generation TKI osimertinib, have been approved by the United States (US) Food and Drug Administration (FDA). Osimertinib benefits NSCLC patients with *EGFR* T790M mutation who fail in first-generation *EGFR*-TKIs treatment (6, 7). However, osimertinib resistances have been reported and *MET* amplification is one mechanism for acquired resistance to third generation *EGFR*-TKIs (8). A previous study indicated that the combinatorial treatment of first/third-generation *EGFR*-TKIs and crizotinib was efficaciously treated two patients with newly acquired *MET* amplification following osimertinib resistance (9). However, *MET* fusion is relatively rare in NSCLC. In this case study, we report a unique LADC case with two uncommon *EGFR* mutations, who had a partial response to icotinib plus crizotinib, with an acquired novel *MET* fusion (*CUX1* exon14-*MET* exon15) following osimertinib treatment. Tolerance for the addition of crizotinib to the *EGFR*-TKI treatment was quite good and proceeded without the emergence of unexpected toxicities.

## Case presentation

**Figure 1:**
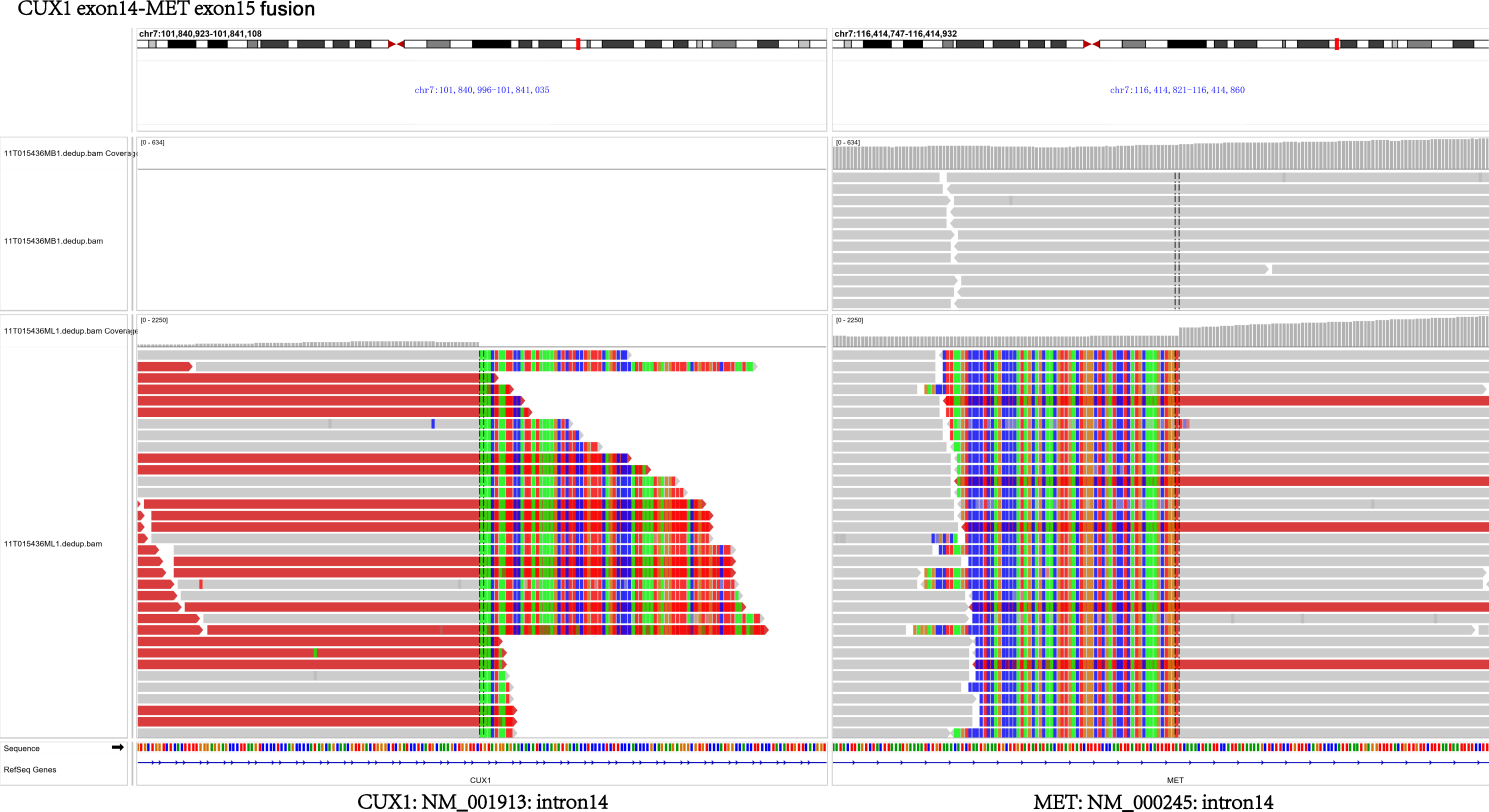
Next-generation sequencing detected *MET* fusion.

**Figure 2:**
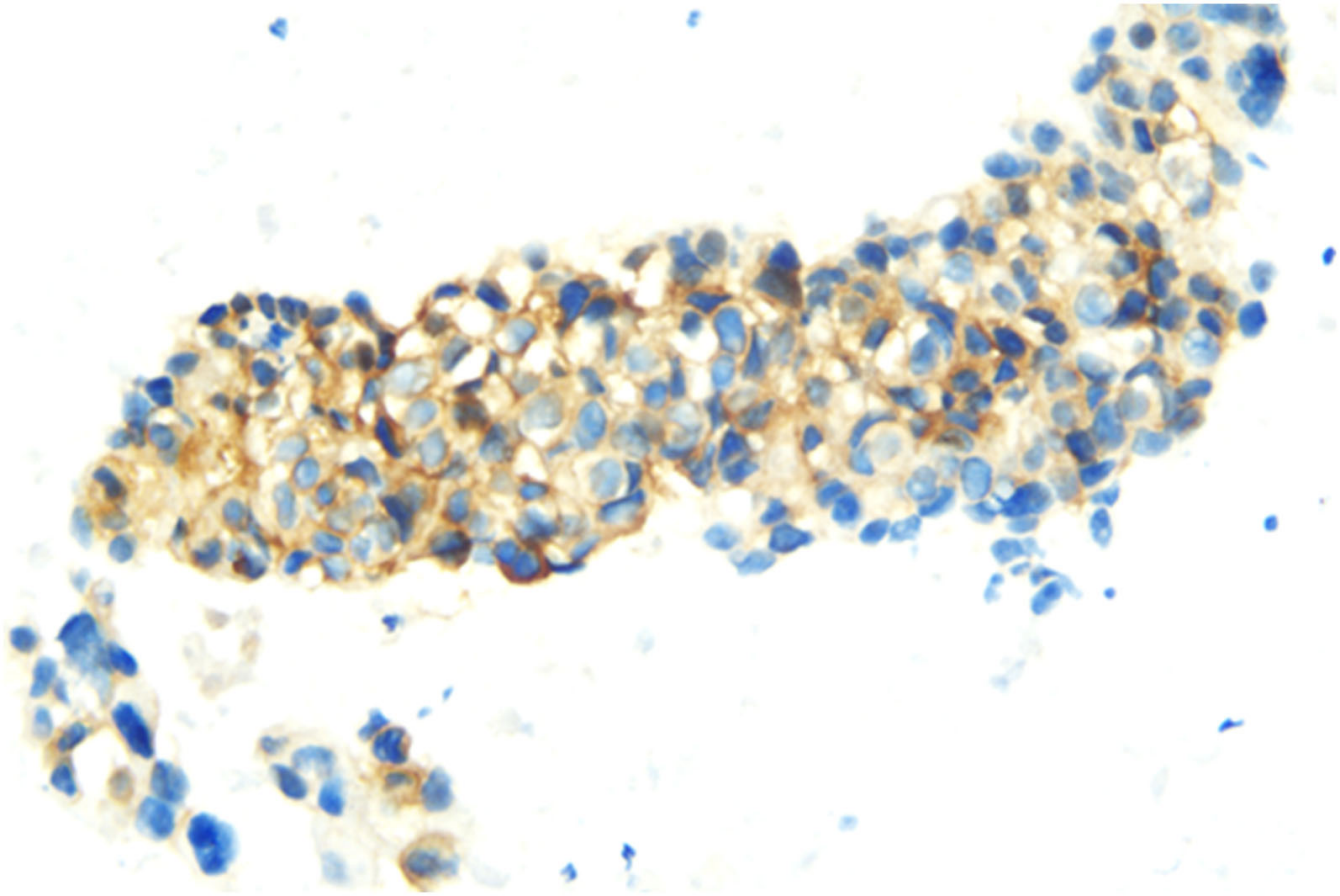
Immunohistochemical staining of MET showing positive in LADC biopsy specimens at 40x.

**Figure 3:**
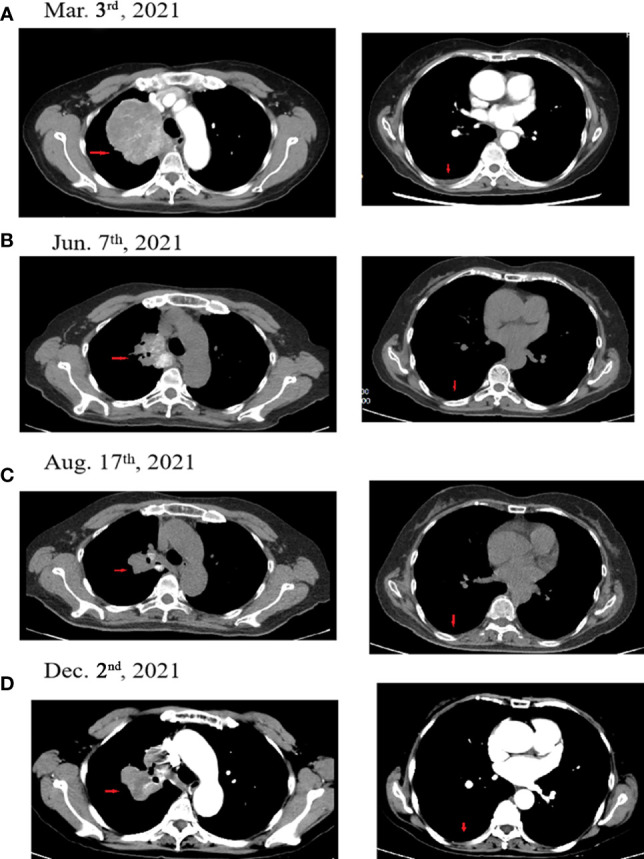
Computed tomographic (CT) images for the patient during the treatment course. **(A)** The patient experienced progressive disease during icotinib therapy (March 3, 2021). **(B)** The patient achieved PR following icotinib plus crizotinib therapy (June 7, 2021). **(C)** The patient achieved PR following icotinib plus crizotinib therapy (August 17, 2021). **(D)** The patient achieved SD following icotinib plus crizotinib therapy (December 2, 2021). Red arrows indicate the tumor.

**Figure 4:**
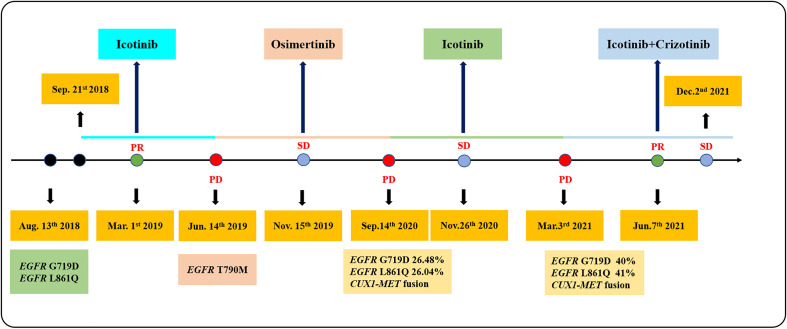
A summary of the patient’s treatment.

In July 2018, a 63-year-old woman, who had never smoked, presented with a cough and sputum, and was diagnosed with LADC (Stage cT4N2M0 IIIB). Biopsy tissue was sent for next generation sequencing (NGS) testing, and two *EGFR* uncommon mutations (exon 21 L861Q and exon 18 G719D) were identified. The patient received the first-generation *EGFR*-TKI icotinib, at a dose of 125 mg, a TID standard daily dose on September 21, 2018, and had a good response upon first evaluation on March 1, 2019. A computed tomographic (CT) scan revealed that the paramediastinal mass, located within the right upper lung, was significantly smaller than previously determined, and the patient achieved PR according to RECIST version 1.1. The patient was re-examined on June 14, 2019 and discouraging information was determined. The CT scan performed at this time indicated an enlarged pulmonary mass and the patient experienced progressive disease. NGS testing was performed again and an *EGFR* T790M point mutation was identified. The patient was then treated with the third-generation *EGFR*-TKI osimertinib. A chest CT on November 15, 2019 revealed disease stability. On September 14, 2020, the CT scan showed an enlargement of lung lesions and the patient, once again, experienced disease progression. An NGS test indicated that in addition to the coexistence of the *EGFR* L861Q/G719D double mutations, a novel acquired *CUX1* (exon 14)-*MET* (exon 15) fusion was identified for the first time (**Figure 1**). An immunohistochemistry assay indicated strong expression for MET (**Figure 2**). From September 14, 2020, the patient was, once again, treated with icotinib (125 mg, TID), and a chest CT on November 26, 2020 revealed disease stability. The patient again experienced disease progression on March 3, 2021 that included the presence of an enlarged lung lesion (74 mm × 78 mm), pleural thickening, and a small amount of pleural effusion (**Figure 3A**). NGS results, concurrent with *EGFR* double uncommon mutations and a *CUX1*-*MET* fusion, led us to a combined targeted strategy with icotinib (125 mg, TID) and crizotinib (250 mg, BID), inhibitors that targeted *EGFR* and MET, respectively, from March 3, 2021. On June 7, 2021, a CT scan revealed that the paramediastinal mass within the lung was significantly smaller (45 mm × 42 mm) than previously determined, and the patient reached PR (**Figure 3B**). To date, nine months have passed, and the patient is still at stable disease (SD) (**Figures 3C, D**). **Figure 4** provides a timeline that includes relevant care data.

## Discussion

*EGFR*-TKIs are recommended for patients with NSCLC having *EGFR* mutations (10). Data regarding treatment effectiveness for *EGFR*-TKIs in NSCLC patients with non-resistant, uncommon *EGFR* mutations are limited. A previous report suggested that the combination of two uncommon mutations is associated with poorer prognosis (median progression free survival (PFS): 6.5 months), as compared to combinations of two common mutations (median PFS: 10.1 months) or a common and uncommon mutation (median PFS: 10.5 months) (11). Substitution mutations G719X, L861Q, and S768I are of particular interest as uncommon mutations. According to a recent molecular epidemiological study in Asia, these three mutations constitute approximately 6% of all *EGFR* mutations (12). The G719X and L861Q mutations were found to be included in several commercial *EGFR* mutation detection kits. These uncommon mutations are occasionally found in daily clinical practice, although the clinical effectiveness of *EGFR*-TKIs in treating patients with these uncommon mutations remains unclear. The response rate to an *EGFR*-TKI treatment has been determined to be significantly higher in tumors with compound mutations (G719X + L861Q and G719X + S768I) as compared to tumors with single mutations (uncommon) (68.4% vs. 37.8%, p = 0.011) (12). Jin et al. (13) reported that icotinib is an effective treatment option for patients with the LADC harboring compound *EGFR* L858R and an A871G mutation (13). Another study reported a case of LADC patient with the same *EGFR* L861Q and R776H compound mutations, who received gefitinib and achieved stable disease (SD) with a PFS of 2.2 months (14). Here, we report a LADC patient with uncommon *EGFR* G719D/L861Q mutations who was treated with icotinib therapy. After five months, the patient achieved PR. However, the patient experienced progressive disease after another three months. To date, the most common acquired resistance mechanism to first generation *EGFR*-TKIs is the T790M mutation, which accounts for approximately 60% of resistance cases (15). With the exception of the T790M mutation (16), optimal treatment for the various mechanisms associated with acquired resistance is not yet clearly defined. Several studies have comprehensively explored the mechanisms of acquired resistance to *EFGR*-TKIs using re-biopsy tissue specimens. In such studies, the most common acquired resistance mechanisms have been attributed to target gene modification, alternative pathway activation, and histological or phenotypic transformation (16).

Many past studies have shown that *MET* alteration is a mechanism of acquired resistance to *EGFR*-TKIs. As a proto-oncogene, the carcinogenic effect of *MET* has been demonstrated for multiple tumors (17). Studies have revealed that mutations within the splice site of *MET* that result in the skipping of exon 14 is important molecular driver for NSCLC (18). *MET* inhibitors have resulted in a therapeutic response in patients with LADC (19). High-level *MET* amplification, *MET* exon 14 skipping alterations, or MET overexpression are different types of *MET* alternations (20). Since *EGFR* mutations and *MET* fusions are markedly sensitive to *EGFR*-TKIs and crizotinib, respectively, clinicians should pay more attention to the existence of *EGFR* mutation and *MET* fusion (21–23). Crizotinib is a small-molecule tyrosinase inhibitor that effectively inhibits high-level *MET* amplification or the *MET* exon 14 skipping mutation. National Comprehensive Cancer Network (NCCN) guidelines have approved crizotinib as a first-line therapy or as a subsequent therapy option for patients with metastatic NSCLC harboring *MET* exon 14 skipping mutation. Cohort studies of osimertinib resistance mechanism, especially MET-associated, have been poorly investigated. NSCLC patient achieved PR to the combination of first-generation EGFR-TKI icotinib and crizotinib, after the previous treatment history of third-generation TKI osimertinib resistance (9). Preclinical studies have suggested that resistance to third-generation EGFR-TKIs would primarily owing to the additional mutations in EGFR itself (for example, C797S) (24, 25). *MET* fusions are rare in LADC. As such, standard treatment for patients with *MET* fusions has not been determined. In past studies, acquired *MET* fusions (*CAV1*-*MET* fusion, *MET*-*UBE2H* fusion, and *SPECC1L*-*MET* fusion) have been identified in three LADC patients with *EGFR* L858R mutation/*EGFR* 19 exon mutations following *EGFR*-TKIs; and a partial reaction to crizotinib was determined (21, 26, 27). To our knowledge, no reports currently exist for LADC patients harboring *EGFR* two uncommon mutations and an acquired *MET* fusion. The *EGFR* mutations reported in previous studies in NSCLC patients with acquired *MET* fusions were common variants. Here, we reported a unique case of LADC patient with *EGFR* double uncommon mutations and an acquired novel *MET* fusion (*CUX1* exon14-*MET* exon15) following icotinib resistance who had a PR to icotinib plus crizotinib administration. A previous report showed LADC patient harboring *MET*-*ATXN7L1* fusion obtained a PR to crizotinib although the disease progressed after four months (22). Another study reported an acquired *MET* fusion following osimertinib therapy in a LADC patient with *EGFR* mutation, however, at the time of the study, the patient had only received therapy for one month and the patient’s condition was significantly declining (21). Therefore, distinguishing the *MET* fusion partners when the MET inhibitor crizotinib is used in clinical practice is very important. Our patient was treated using a combined targeted strategy of icotinib and crizotinib, and reached PR. To date, nine months have passed, the patient remains at SD.

In conclusion, we reported a unique case of LADC patient with *EGFR* double uncommon mutations and an acquired novel *MET* fusion (*CUX1* exon14-*MET* exon15) following treatment with osimertinib that had a PR to the administration of icotinib plus crizotinib. Here, for the first time, we suggest that *MET* fusion may be one of the resistance mechanisms in patients with *EGFR* uncommon mutations and that distinguishing *MET* fusion partners is important for crizotinib treatment.

## Data availability statement

The raw data supporting the conclusions of this article will be made available by the authors, without undue reservation.

## Ethics statement

This study has been approved by ethics committee of The First People’s Hospital of Foshan. Written informed consent for participation was not required for this study in accordance with the national legislation and the institutional requirements. Written informed consent was obtained from the individual(s) for the publication of any potentially identifiable images or data included in this article.

## Author contributions

WF, LO, YT, and YD all participated in the management of this case. LG, QH, and TH were in charge of data collection. WF and LO did the modification. All authors contributed to the article and approved the submitted version.

## Acknowledgments

We thank the patient and her family for their engagement in this case study.

## Conflict of interest

Authors LG, QH, and TH were employed by Shanghai OrigiMed Co., Ltd.

The remaining authors declare that the research was conducted in the absence of any commercial or financial relationships that could be construed as a potential conflict of interest.

## Publisher’s note

All claims expressed in this article are solely those of the authors and do not necessarily represent those of their affiliated organizations, or those of the publisher, the editors and the reviewers. Any product that may be evaluated in this article, or claim that may be made by its manufacturer, is not guaranteed or endorsed by the publisher.
